# Diagnosis of Fetal Alcohol Spectrum Disorders (FASDs): Guidelines of Interdisciplinary Group of Polish Professionals

**DOI:** 10.3390/ijerph18147526

**Published:** 2021-07-15

**Authors:** Katarzyna Okulicz-Kozaryn, Agnieszka Maryniak, Magdalena Borkowska, Robert Śmigiel, Katarzyna Anna Dylag

**Affiliations:** 1 Institute of Mother and Child, 01-211 Warsaw, Poland; katarzyna.okulicz@imid.med.pl; 2 State Agency for Prevention of Alcohol-Related Problems, 02-326 Warsaw, Poland; magdalena.borkowska@parpa.pl; 3 Faculty of Psychology, University of Warsaw, 00-183 Warsaw, Poland; agnieszka.maryniak@psych.uw.edu.pl; 4 Department of Pediatrics, Division of Propaedeutic of Pediatrics and Rare Disorders, Wroclaw Medical University, 50-368 Wroclaw, Poland; robert.smigiel@umed.wroc.pl; 5 St. Louis Children Hospital, 31-503 Krakow, Poland; 6 Department of Pathophysiology, Jagiellonian University Medical College, 31-121 Krakow, Poland; 7 Department of Pediatrics, Andrzej Frycz Modrzewski Krakow University, 30-075 Krakow, Poland

**Keywords:** fetal alcohol spectrum disorders, FASD, diagnosis, guidelines, Polish recommendation

## Abstract

(1) Background: Considerable prevalence in Poland and serious health consequences of prenatal alcohol exposure indicated the need to develop national guidelines for the diagnosis of fetal alcohol spectrum disorders (FASDs). It was assumed that the guidelines must be in line with international standards but adjusted to the Polish context. (2) Methods: Work on recommendations was carried out by an interdisciplinary team of Polish specialists. Its first stage was to assess the usefulness in our country of the U.S. and Canadian guidelines. In the second stage, after several rounds of discussions, a consensus was achieved. (3) Results: The Polish guidelines for diagnosing FASD cover the following issues: 1. distinguished diagnostic categories; 2. diagnostic procedure; 3. assessment of prenatal exposure to alcohol; 4. assessment of sentinel facial dysmorphias; 5. assessment of body weight, height, and head circumference; 6. neurodevelopmental assessment. An important element of the recommendation is appendices containing practical tools that are useful in the diagnostic procedure. (4) Conclusions: National guidelines may improve the quality and standardization of FASD diagnosis in Poland, but their practical utility has to be monitored.

## 1. Introduction

Fetal alcohol spectrum disorders (FASDs) are a group of neurobehavioral conditions caused by prenatal alcohol exposure (PAE). The prevalence of FASD in the general pediatric population worldwide has been estimated at 7.7 cases per 1000 (95% CI, 4.9–11), with the highest rates (19.8 per 1000; 95% CI, 14.1–28.0) in the European region [1]. Even more alarming estimated prevalence was reported by Roozen at al. [2]. In Poland, the incidence of FASD is not less than 20 cases per 1000, while the incidence of fetal alcohol syndrome (FAS) is not less than 4 per 1000 [3]. FASD is characterized by significant comorbidity including congenital malformations as well as intellectual and behavioral disorders [4]. In contrast to most medical conditions, there is no “gold standard” of FASD diagnosis, neither is there a single biochemical or imaging test that can determine whether an individual has FASD. Therefore, since 1996, the diagnosis of FASD has been established based on clinical criteria [5]. Several recommended diagnostic systems for FASD diagnosis exist parallelly in different parts of the world [6,7,8], and work is still underway to develop models that best suit the needs of individual countries [9,10]. It is worth mentioning that the existing diagnostic systems, although validated, vary significantly, which leads to serious misunderstandings and confusion [11].

In Poland, there are currently over a dozen facilities specializing in working with people with FASD, and many others provide services in this area as one of the elements of a wider healthcare offer [12]. Most of them use their own diagnostic criteria, usually, although to a varying degree, based on one of the three best known from the literature guidelines [6,7,8]. Therefore, FASD diagnoses from one center/organization are sometimes questioned by another center/organization resulting in patients’ and their parents’ confusion and delay in provision of adequate therapy. Moreover, the quality of diagnosis across centers is generally unknown and does not reflect the growing research evidence in the area of FASD.

Agreeing on FASD diagnostic standards that could be accepted by the majority of representatives of such a diverse environment requires good understanding and open discussion in the widest possible group of professionals. This was the key assumption made by the team affiliated by the State Agency for Prevention of Alcohol-Related Problems initiating the process of elaboration of Polish guidelines to diagnose FASD in 2018.

The second assumption was that Polish recommendations should be compatible with FASD diagnostic guidelines that are most frequently mentioned in the world literature. In general, the solutions proposed in each of these systems are mostly based on the same research, take into account the same areas of diagnosis, and adopt similar criteria. However, in detail, there are significant differences between them which lead to a different classification of the same cases [8,13]. All three above-mentioned diagnostic guidelines have been developed and tested in the North American context, significantly different from reality in Poland concerning the healthcare system. Therefore, probably none of them can be transferred directly to Poland.

Based on these assumptions, Polish recommendation for FASD diagnosis were elaborated, and the aim of this article is to present the guidelines for FASD diagnosis developed by an interdisciplinary group of Polish professionals.

## 2. Materials and Methods

### 2.1. Work on the FASD Diagnostic Recommendations Included Several Steps

#### 2.1.1. Step 1. Formation of a Team of Professionals

**Figure 1:**
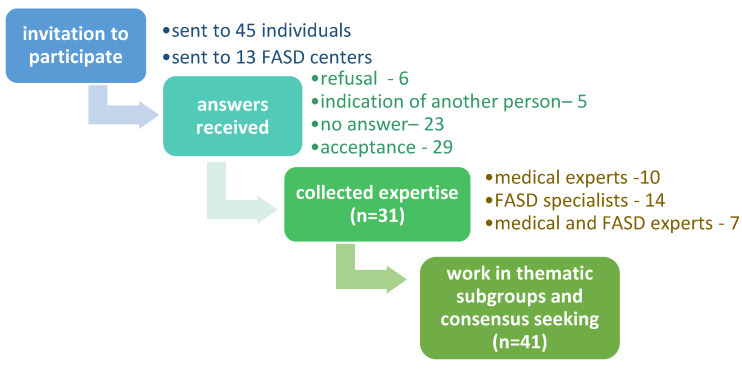
Scheme of creating a team of authors of Polish FASD diagnostic recommendations.

Invitations to cooperate were sent to all institutions dealing with FASD in Poland. Only a few that had been in existence for less than a year, were excluded due to the limited experience of their staff in the field of FASD. Individual invitations were addressed to specialists in medical and related sciences, such as genetics, gynecology, neonatology, pediatrics, neurology, psychiatry, psychology, and speech therapy. Out of 58 invitations, positive responses were received in 29 cases and refusals to cooperate in 6 cases (Figure 1). In the first round of consultation consisting in collecting questionnaire opinions, responses were received from 31 specialists. During further rounds of consultations, more people joined the team of professionals, invited by project coordinators due to their specialist knowledge in the field and underrepresented in the original team composition, or invited in the first stage, but who then did not cooperate.

#### 2.1.2. Step 2. Gathering the Opinions of Professionals on the Three (U.S./Canadian) FASD Diagnostic Systems

The first round of consultations was inspired by the AGREE II procedure (Appraisal of Guidelines, Research, and Evaluation, version II). It is a tool that facilitates the assessment of the methodological quality and transparency of the proposed standards of operation in the area of healthcare [14] and covers the following areas:

1. scope and purpose of the recommendation,
2. scope of consultation,
3. methodological rigor of developing recommendations,
4. clarity of presentation,
5. utility,
6. authors’ independence, and
7. overall assessment.

In our project, the original procedure has been modified in order to facilitate the most reliable comparisons between the recommendations formulated in each FASD diagnostic guideline [6,7,8]. The aim was to determine, based on the collected professionals’ opinions, which of the recommendations are most useful in Poland. The evaluated recommendations concerned the following:

1. featured categories/units under the FASD term,
2. diagnostic procedure,
3. assessment of prenatal exposure to alcohol,
4. assessment of neurodevelopmental disorders,
5. assessment of key facial dysmorphias,
6. assessment of height/weight, and
7. formulating the diagnosis and the action plan.

The evaluation criteria for each of these recommendations included the following:

- clarity of presentation,
- methodological rigor in developing recommendations,
- utility in Poland, and
- overall assessment.

For each recommendation, 9 to 15 questions were formulated with a 5-point scale of answers, from “I strongly disagree” to “I strongly agree” and a space for comments (a sample question is presented in the Table 1). Questions regarding a given recommendation were preceded by a description of the recommendation in each of the three guidelines.

#### 2.1.3. Step 3. Four Rounds of Consultation and Work in Subgroups

The professionals’ opinions on the U.S./Canadian guidelines were the starting point for team work on Polish diagnostic standards. A joint analysis of the strengths and weaknesses of the existing recommendations made it possible to define the scope and general schedule of further work of the team divided into thematic groups working on the proposals for recommendations regarding a specific area of diagnosis:

- Group 1—recommendations for neurodevelopmental assessment,
- Group 2—recommendations regarding prenatal exposure to alcohol,
- Group 3—recommendations for the diagnostic procedure, and
- Group 4—recommendations for the assessment of growth and dysmorphia.

It was decided that the recommendations for recognition of specific categories within FASD will be formulated after reaching the consensus on the recommendations in other areas.

In total, there were four rounds of consultations summarized by panel meetings (including one online meeting due to the COVID-19 pandemic).

#### 2.1.4. Step 4. Adoption of the Recommendation

The last panel meeting (June 2020) was aimed at the passing of the recommendations as ready to be published and disseminated. Agreed recommendations were accepted by acclamation and published in the special issue of Medycyna Praktyczna-Pediatria (2020) [15].

The project to develop FASD diagnostic standards was entirely financed by the State Agency for Prevention of Alcohol-Related Problems (PARPA).

## 3. Results

### 3.1. Evaluation of U.S./Canadian FASD Diagnostic Guidelines

**Figure 2:**
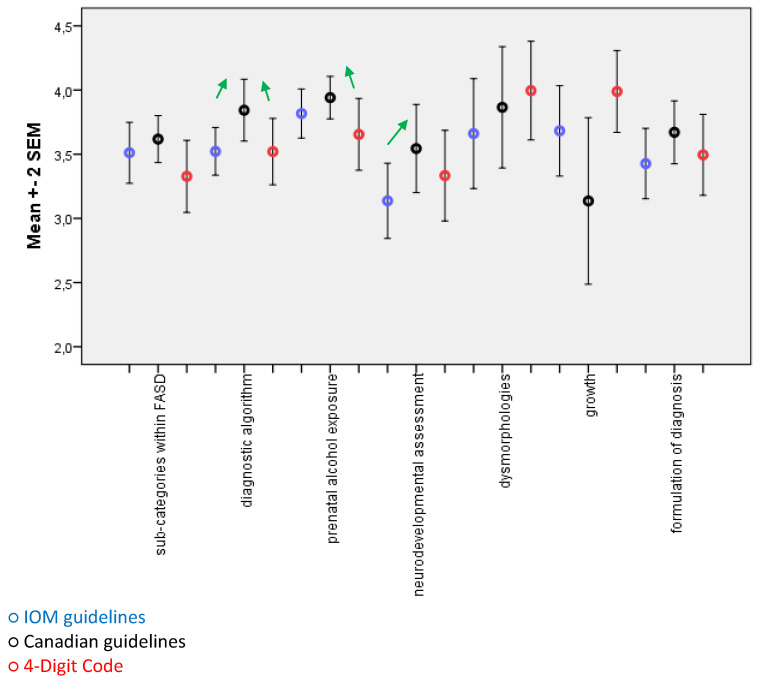
The overall assessment means scores and standard errors of IOM (blue), 4-Digit Code (red), and Canadian (black) guidelines to diagnose FASD (statistically significant differences are indicated by the green arrows).

All three diagnostic guidelines [6,7,8] were positively evaluated by Polish professionals with the overall assessment mean scores between 3 and 4 on the 5-point Likert scale (Figure 2). Not all experts assessed all the recommendations (e.g., psychologists did not feel competent to assess issues related to growth, and people working on the basis of the 4-Digit Code did not want to evaluate the IOM and Canadian recommendations). Therefore, the number of collected assessments ranged from 11–13, in the case of recommendations regarding the assessment of dysmorphias and growth, to 29–31 in the case of the diagnostic algorithm. Still, statistically significant differences (*t*-test for paired samples) were observed in some categories, in favor of the Canadian guidelines [7]. This received higher scores than IOM guidelines (*t* = 2.128; *p* = 0.042) and a 4-Digit Code (*t* = 2.198; *p* = 0.036) in the “diagnostic algorithm” category; higher than the 4-Digit Code (*t* = 2.560; *p* = 0.019) in “prenatal alcohol exposure” assessment and higher than IOM [6] (*t* = 3.105; *p* = 0.006) in “neurodevelopmental assessment”. The differences in ratings of other categories did not achieve statistical significance (*p* > 0.05) although, as shown in Figure 2, the exclusion of the growth criterion from the Canadian diagnostic recommendations was very critically received by Polish specialists.

**Figure 3:**
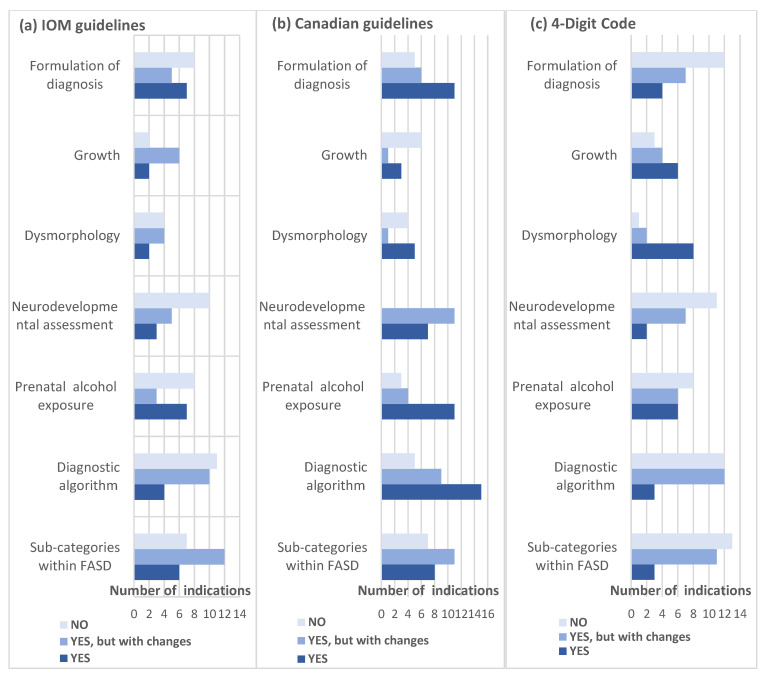
Professionals’ answers to the question of whether each of the recommendations should be implemented in Poland (“no”, “yes, but with some changes”, “yes”). (**a**) IOM guidelines [6]; (**b**) Canadian guidelines [7]; (**c**) 4-Digit Code [8].

Professionals’ answers to the question whether a given recommendation should be introduced in Poland provided further indication for the team’s work (Figure 3). These suggested to base the work on the recommendations on the diagnostic algorithm and procedure of diagnosis formulation, as well as on the assessment of the prenatal alcohol exposure and neurodevelopmental assessment mainly on the Canadian guidelines [7], perceived as the most applicable in our country. The IOM guidelines [6], as shown by the qualitative answers, were perceived by some Polish practitioners as too academic and hard to apply, and therefore, the need for their further adaptation to the national context was suggested. The 4-Digit Code [8] was evaluated as the most useful in terms of growth and dysmorphia assessments.

The conclusions from the evaluation of U.S. and Canadian FASD diagnostic guidelines might be summarized as follows:

- Dysmorphology and growth assessment are not very controversial. The key task is to select growth charts to be used in Poland, as at present, the practice in this area significantly differs across specialists and diagnostic centers.
- The research evidence on the usefulness of various methods to assess prenatal alcohol exposure among Polish women of childbearing age and during pregnancy is missing.
- In-depth understanding of biological mechanisms and factors determining structural and functional development of the central nervous system is needed to develop standards of neurodevelopmental assessment.
- The neurodevelopmental assessment is probably the most challenging element of FASD diagnosis. However, the general consensus was achieved that the scope of assessment has to be broad—covering all cognitive, emotional, and social functions, acknowledging environmental factors (family, school).
- In the area of neurodevelopmental assessment, further discussion is needed to determine:
  ■ selection of psychological and neuropsychological tools (the first list of validated tests will be elaborated by the work group soon),
  ■ selection of the cutoff point (1.5 or 2 SD) for test results,
  ■ interpretation of the general IQ scores,
  ■ whether other neurodevelopmental disorders should be recognized in a patient with FASD or whether an individual cognitive profile should be prepared, and
  ■ inter-sectoral cooperation of mental health specialists (between education and healthcare systems) and educational judicature (How psychologists working out of the healthcare system should be involved in FASD diagnosis?).
- In Poland, a three-stage diagnostic algorithm (including basic screening in primary healthcare, social-care, and educational units; proper assessment by the interdisciplinary team; consultations with a highly specialized medical center for genetic, neuroimaging, and neurometabolic assessment) might be useful.

### 3.2. Polish Guidelines for Diagnosing Fetal Alcohol Spectrum Disorders

The Polish guidelines for diagnosing FASD, include six chapters:

1. distinguished diagnostic categories,
2. diagnostic procedure,
3. assessment of prenatal exposure to alcohol,
4. assessment of sentinel facial dysmorphias,
5. assessment of body weight, height, and head circumference, and
6. neurodevelopmental assessment.

An important element of the recommendation is appendices containing practical tools that are useful in the diagnostic procedure.

#### 3.2.1. Distinguished Diagnostic Categories

Polish professionals indicated several limitations of currently used terminology in the area of FASD. They argued that terms used in the IOM diagnostic guidelines [6] such as alcohol-related birth defects (ARBDs) and alcohol-related neurodevelopmental disorder (ARND) [16] are not based on current scientific knowledge and suggest a random cause and effect relationship between alcohol exposure and symptoms. Moreover, according to the IOM guidelines, it is difficult to diagnose ARND in children under 3 years of age, which may delay the initiation of the therapeutic process. On the other hand, the categories distinguished in the 4-Digit Code [8] are overly detailed, which is not conducive to diagnostic clarity. It creates confusion among patients, their family members/caregivers, and those working with a patient. Moreover, such a detailed distinction is of little importance when developing post-diagnosis recommendations. The Canadian guidelines [7] were perceived by some specialists as too simplistic. Although the “at risk” category was accepted with general approval as giving space to observe the child’s development and re-diagnose.

Ultimately, it is recommended to distinguish the two basic diagnostic categories within FASD (Table 2):

- FAS (Q86.0 in the ICD-10 classification)
- ND–PAE (neurodevelopmental disorder associated with prenatal alcohol exposure)—although ND-PAE is not recognized in the ICD-10 classification, it is recommended to register this as G96.8: Other specified disorders of the central nervous system.

This recommendation arose from the specificity of the Polish healthcare system in which the amount of service provision is calculated based on the ICD-10 diagnosis the provider reports. Therefore, other ICD-10 diagnostic categories that are most often used by Polish providers to describe symptoms observed in ND-PAE are also indicated: F80—Specific developmental disorders of speech and language; F81—Specific developmental disorders of scholastic skills; F82—Specific developmental disorder of motor function; F83 Mixed specific developmental disorders; F88—Other disorders of psychological development; F94—Disorders of social functioning with onset specific to childhood and adolescence; E34.3—Short stature, not elsewhere classified; R27—Other lack of coordination; R62—Lack of expected normal physiological development; R62.8—Other general symptoms and signs.

Patients undergoing the diagnostic process requiring further assessments can be classified as “at risk” of FASD.

#### 3.2.2. Diagnostic Procedure

Polish professionals generally agreed that the differences between U.S. and Canadian diagnostic guidelines largely reflect differences between health systems and different patient care logics and that the same national context and practice-related considerations should guide development of Polish recommendations. Therefore, Polish recommendations had to be constructed adequately to the following facts:

- FASD is in general underdiagnosed especially among individuals staying in biological families.
- The doctor has very little time for the patient (the regulations set by the National Health Fund limit consultations to 20 min, including the time devoted to administrative duties).
- The diagnosis once entered into the patient’s file is rarely verified by consecutive doctors. Most often, the consecutive consultants of the patient prescribe the previous diagnoses and add their own.
- The current system of financing outpatient specialist care by the National Health Fund does not create the possibility of organizing multispecialist consultations and referring the patient to other specialists, radically extends the process of diagnosis (queues to specialists). Multispecialist consultations are most often offered by private or public centers thanks to the funds from other sources—e.g., municipal budgets.

**Figure 4:**
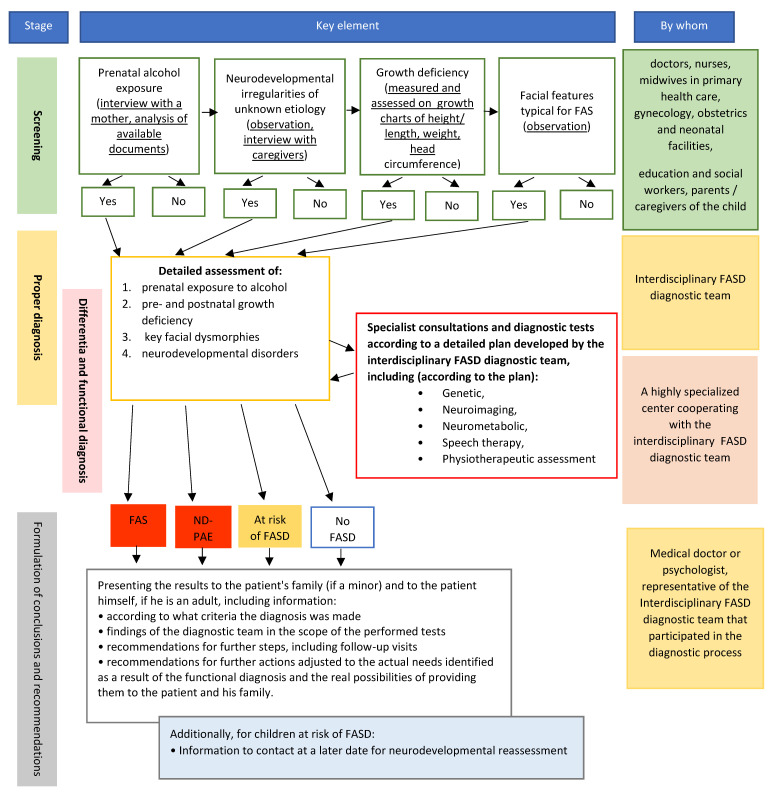
Recommended FASD diagnostic procedure.

Therefore, Polish diagnostic recommendations should take into account the possibility of cooperation of a wide range of people working with children and adolescents in order to facilitate early recognition of warning signals that may suggest disorders from the FASD group. At the same time, it should be emphasized that the proper diagnosis should be made by the professional, interdisciplinary team. Finally, recommended diagnostic procedure consists of four stages (Figure 4):

(1) Screening—to enhance early identification of risk and referrals to the proper diagnosis of FASD.
(2) Proper diagnosis—to check whether a person meets the FAS or ND–PAE diagnostic criteria, or whether he/she should be classified as at risk of FASD; to formulate functional diagnosis; and to indicate the necessary tests to complement the differential diagnosis.
(3) Differential and functional diagnosis—to rule out causes of neurodevelopmental disorders other than prenatal exposure to alcohol, and thereby ultimately confirm a diagnosis of FAS or ND–PAE. It may also exclude a patient from the FASD risk group.
(4) Formulation of conclusions and recommendations and their presentation to a patient and/or family/caregivers.

It is recommended to screen all children whose mothers drank alcohol during pregnancy; with neurodevelopmental disorders of unknown etiology; with impaired growth (length/height and/or body weight less than the 3rd percentile and/or birth weight less than the 10th percentile and/or microcephaly); with facial dysmorphologies characteristic of FAS.

Screening tests can be carried out by different specialists and at different levels of institutional healthcare system, in particular:

- In obstetrics and pediatric clinics—based on an interview with the mother or other data indicating alcohol consumption during pregnancy.
- In obstetrics and neonatal departments—based on the observation of neurological disorders or congenital abnormalities in the newborn, including dysmorphia.
- By pediatricians or nurses, in various healthcare facilities—based on the observation of any neurodevelopmental abnormalities or growth restriction or dysmorphia in the child (Tools supporting professionals in observing feeding problems in a child under 2 years of age and approximate assessment of the functions of the nervous system for non-physicians in order to make a decision about referring a child over 2 years of age to a neurologist are included, as appendixes, in the Polish recommendations).

Moreover, education or social workers, or parents/guardians of the child should be aware that the observation of disorders in their psycho-social functioning or difficulties in the implementation of developmental tasks may be a signal to refer to the specialists (The short questionnaire to guide observation of child’s behaviors that may be a signal to refer to the diagnosis toward FASD, is included).

Currently, standardized tools for FASD screening are not available. Therefore, in the screening phase, the attentiveness and knowledge of GPs, nurses, midwives, social workers, and psychologists are crucial. It depends on them whether and when the process of specialist diagnosis toward FASD will be launched.

Due to the complexity of the health consequences of prenatal alcohol exposure, at the stage of proper diagnosis, an interdisciplinary team of specialists is essential for the accurate and comprehensive diagnosis of FASD and for the development of therapeutic recommendations. The core of each team is a pediatrician or family doctor, as well as a clinical psychologist or neuropsychologist, and in the case of infants (up to 18 months of age), also a pediatric neurologist and a speech therapist. All team members should have the necessary expertise to conduct the assessment in all necessary areas. They should also receive training in obtaining sensitive information from biological families, especially on prenatal exposure to alcohol.

In order to exclude other diseases, in the case of existing indications, the FASD diagnostic team may refer a patient for a genetic, neuroimaging, or neurometabolic test. At this stage, it is necessary to include a geneticist in the diagnostic team.

An indispensable element of functional diagnosis is in-depth examinations by a speech therapist (in Polish recommendations described in a separate appendix) and physiotherapist (also described in an appendix).

The results of the assessment should be presented to the minor patient’s family and to the patient himself, if he is an adult. In the case of adolescents, the diagnostic team has to decide for themselves how to present the results of the diagnosis to them.

The results of the FASD diagnosis process should be communicated to the patient and/or their parents/guardians personally, based on the written opinion containing the following information:

- according to what criteria the diagnosis was made;
- findings of the diagnostic team in the scope of the performed tests (assessments);
- recommendations for further steps, including follow-up visits (it must be adjusted to the actual needs identified as a result of the functional diagnosis and the real possibilities of providing them to the patient and their family); and
- in the case of children at risk of FASD—information about the need to contact the diagnostic team at a specific time for the reassessment as well as information on the areas of the child’s functioning that should be observed by parents/caregivers in the meantime.

It is also recommended to ensure a clear and simple form of communication and to encourage the patient and/or their family to contact the diagnostic team at a later date with additional questions or concerns.

#### 3.2.3. Assessment of Prenatal Exposure to Alcohol

Assessment of maternal alcohol consumption during pregnancy is an integral part of the FASD diagnostic process. It has not yet been established whether there is a safe dose of alcohol for the fetus. The teratogenic effect of alcohol may vary depending on the timing of exposure (the stage of central nervous system development, genetics, and other individual factors) there is no single dose of alcohol that would be equally dangerous in each case [17,18,19,20,21,22]. Therefore, it is crucial to assess the level of alcohol consumption in pregnancy in each case.

Based on the previous studies [23,24,25,26,27,28,29] and taking into account that, in Poland, a standard drink (one dose) is defined as 10 g of pure alcohol, it is recommended to recognized ND–PAE if a woman had approximately

- ≥8 standard alcohol doses per week for ≥2 weeks of pregnancy or
- ≥2 heavy drinking episodes (having on one occasion ≥4 doses of alcohol).

In case of FAS diagnosis, drinking (at the levels described above) may be either confirmed or unknown.

Several sources may be used in order to confirm prenatal exposure to alcohol, including the following:

(1) Direct interview—reliable information can only be obtained from the mother herself if an interviewer is in good contact with her and creates an atmosphere of security and trust. Therefore, it is recommended to
  - introduce questions about drinking alcohol to a broader medical interview (an example of such an interview is provided in the attachments);
  - avoid closed questions, i.e., those to which the patient only answers YES or NO;
  - collect information on frequency, quantity, heavy drinking episodes, and timing of alcohol use during pregnancy [28,30];
  - use a standardized tool—AUDIT-C [19,31]. In patients whose AUDIT-C result indicates an increased risk of prenatal alcohol exposure, it is advisable to complete the interview with the complete the full AUDIT test [32,33]; and
  - asking about the period of 3 months before pregnancy (or learning about pregnancy) may be a better predictor of drinking alcohol during pregnancy than a direct question about drinking alcohol during pregnancy [34].
(2) Indirect interview—based on information from other people who have contact with the mother during pregnancy.
  - It should be remembered that the persons providing the information should be reliable, and there should be no conflict of interest between them and the mother.
  - Indirect information (e.g., about a woman’s lifestyle in general or the use of alcohol in other pregnancies) by itself cannot be taken as a significant indicator of PAE.
(3) Medical, judicial, or employee records as well from broadly understood social welfare
  - documented social or legal problems related to drinking alcohol during pregnancy (e.g., driving under the influence of alcohol); and
  - documented alcohol intoxication during pregnancy (study alcohol content in blood, exhaled air, urine).

This part of the recommendations includes several practical guidelines on collecting information on alcohol use during pregnancy, taking into account that this is an extremely sensitive matter and that women may avoid confessing such socially undesirable behavior.

#### 3.2.4. Assessment of Sentinel Facial Dysmorphias

Facial dysmorphism has been linked with prenatal alcohol exposure since the first scientific reports [35,36]. Although, initially, the authors emphasized more dysmorphic features, the triad of short palpebral fissure, smooth philtrum, and narrow upper lip has been considered pathognomonic for fetal alcohol syndrome. Existing criteria are consistent that the presence of three key dysmorphic features is characteristic for fetal alcohol syndrome [6,7,37]. The authors of the Canadian criteria [7] put all other patients (patients with two to zero key dysmorphic features) under the category “non-dysmorphic FASD”, while Hoyme et al. [6] differentiate pFAS (with two out of three dysmorphic features) from ARND (one or zero dysmorphic features). Moreover, Hoyme et al. recommend the use of “dysmorphology index” to evaluate the presence of other dysmorphic features.

The method used for the evaluation of palpebral fissure remains a controversy, whilst the criteria are in agreement with each other regarding the use of lip–philtrum guide for the evaluation of lip and philtrum. A computerized measurement is preferred by the authors of the Canadian criteria [7]. In the 4-Digit Diagnostic Code [8], the measurement can be performed directly with a ruler or from a photograph, whilst Hoyme et al. [6] suggest the direct, manual measurement with a ruler [6]. The three methods were proven to be inconsistent [38]. The choice of the charts [39] as well as the threshold below which the results are considered abnormal (varying from −2 SD [7] to the 10th percentile) [6] remain controversial.

In our recommendations, we suggest the double measurement of palpebral fissure- direct with the plastic ruler, with an analogous method to the one published by Hoyme et al. [6] and, from a photograph, according to the method published by Astley et al. [40]. The arithmetic average from the four measurements (two eyes with two methods) is considered a representative value. Until the moment the charts for palpebral fissure length for Polish population are published, we recommend the use of Scandinavian charts [41]. The threshold is established on −2 SD, values below this should be considered sentinel for FAS. The measurements in the newborn should be treated with caution. We recommend the direct evaluation of lip and philtrum with the use of a race-specific lip philtrum guide [8]. For patients of Asian origin or specific ethnic groups, special caution should be exercised. The presence of the dysmorphic features of the other aforementioned three sentinel facial features [42] should be recorded in the medical documentation of the patients but does not influence the diagnosis. However, the constellation of dysmorphic features that suggests the comorbidity or differential diagnosis of other genetic conditions indicates the need for further evaluation by clinical geneticists. The list of genetic syndromes for differential diagnosis is published in the appendix of the Polish recommendations.

#### 3.2.5. Anthropometry

##### Growth Impairment

Growth impairment has been considered a principal characteristic of FAS since the first official reports published in medical literature [36,43]. Although the mechanisms in which PAE affects prenatal/postnatal growth remain unclear, there is evidence that supports this association [44,45,46,47,48]. Prenatal and/or postnatal growth impairment has been included in the first IOM criteria from 1996 [5] and in the 4-Digit Diagnostic Code [8].

On the other hand, the authors of the recent version of the Canadian guidelines [7] decided to remove growth impairment from their diagnostic criteria on the basis of one publication [49] in which the authors refer to small gestational age only. This approach was then disputed by Astley at al. [50].

Acknowledging the current evidence for specificity and sensitivity of prenatal or/and postnatal growth restriction in FAS, this feature is included in the Polish diagnostic criteria. According to the recommendations, each height/length and weight recorded from birth to the moment of diagnosis should be evaluated. Historical measurements have to be treated with cautiousness, especially if they were recorded at the moment when confounding factors were present (malnutrition, severe illness, maltreatment). Birth weight small for gestational weight (SGA) or evidence of postnatal growth impairment are considered a positive feature supporting FAS recognition. SGA is interpreted as birth weight below the 10th percentile [51]. Growth deficiency is interpreted as either height or weight below the 3rd percentile for sex/age at any moment during the child’s life. The recommended growth charts are WHO growth charts for children born from term pregnancies [52] and charts created by Fenton et al. for children born prematurely [53].

##### Occipitofrontal Circumference (OFC) Evaluation

Microcephaly is a physical feature associated with PAE [42,54]. It has been estimated that 10.2% of children with FASD have microcephaly [55]. The decreased OFC is always associated with decreased brain volume; however, a decreased brain volume can be observed among individuals with FASD with OFC within the referential norms [55]. Although microcephaly can be considered a part of neuropsychological evaluation, we recommend that it is measured by a qualified physician or nurse according to the manual [56]. Fenton et al. charts [53] should be used initially for children born prematurely. For children from term pregnancies and children born prematurely after 50 weeks of postmenstrual age we recommend using WHO OFC charts [52] for ages 0–5 years and Institute for Mother and Child charts [57] for children older than 5 years. As these charts used were created with different methodology, caution has to be maintained in the transition between the charts to avoid overdiagnosis.

#### 3.2.6. Neurodevelopmental Assessment

One of the symptoms of FASD is cognitive impairment. While the typical FASD profile has not been described so far, the research shows some trends. In children with FASD, deficits in learning and memory, executive functions, and adaptation abilities are widespread, which has an impact on their overall coping with the challenges of everyday life and school education [58,59,60,61]. According to the criteria, to recognize FASD disorders, deficits must be in several areas from language, attention, memory, visual–spatial functions, executive functions, congition of various modalities, academic skills, and general intelligence. Moreover, adaptation difficulties and problems in social functioning are also substantial [6,7].

Based on the literature and their own clinical experience, the authors of the standards decided that four areas were included in the neurodevelopmental assessment:

- cognitive functions (gnosis, praxis, attention, language and communication, visual-spatial functions, memory and learning, executive functions, graphomotor skills, general intelligence);
- emotional and social functioning (adequacy and compassion of emotions, mentalization ability, theory of mind, understanding and observance of social norms, implementation of developmental tasks, relationships with peers);
- adaptive difficulties (physiological processes: sleep, eating, symptoms of the autonomic nervous system, self-regulation processes, sensory sensitivity); and
- psychopathological symptoms (anxiety, behavioral disorders, personality development disorders).

It was concluded that to meet the criterion of neurobehavioral impairment, it is necessary to recognize the following:

1. The presence of deficits in at least three cognitive areas. In the case of neurological symptoms—deficits in two areas.
2. The occurrence of abnormalities in at least three areas from the emotional and social sphere, adaptation disorders, or psychopathological symptoms.
3. Significant impact of the identified deficits and symptoms on everyday life activities and school functioning (in the case of people who have completed their education, the data from the interview are to be taken into account).

This part of the recommendations emphasizes the importance of the child’s environmental factors and experiences on the neuropsychological examination results. The authors added the recommendation: when the facts about past traumatic experiences, significant neglect, and environmental deprivation are known, caution should be exercised in diagnosing and assessing deficits. In addition, more frequent follow-up should be suggested. In periods of major changes in the child’s social (e.g., care) or health situation, it is advisable to re-examine the child after 6 months.

The recommendations include suggestions for repeating control tests, the scope of which should be adapted to a person’s needs, identified dysfunctions, and possible new problems reported. It was recommended that follow-up examinations be performed (if the diagnosis was made earlier):

- at 4 years of age;
- about 6–7 years of age (in Poland, this is the age of starting school education);
- around 11–12 years of age; and
- around 18 years of age.

This part of the recommendations was supplemented with three appendices: a list of standardized neuropsychological methods with current Polish standardization, a scheme of speech-therapist examination, and a description of a physiotherapeutic examination.

## 4. Discussion

Polish diagnostic guidelines were created to improve the quality of FASD diagnoses and standardize the criteria used in various existing centers as well as to give clear guidelines to the future ones. Only in a few years will it be possible to assess to what extent these goals have been achieved. However, we can already say that the process of developing the recommendations contributed to the integration of specialists dealing with FASD issues and allowed to start a substantial discussion on the current methods of operation and factors facilitating and limiting the recognition of FASD. Another success is the tightening of cooperation between representatives of the medical community and non-governmental organizations (NGOs), which have been the most active in Poland in the field of FASD for years.

A very important stage in the process of developing Polish recommendations was the assessment of the American and Canadian guidelines for the diagnosis of FASD [6,7,8]. This allowed us to focus the discussions on the available scientific evidence supporting different solutions and not on the individual clinical experiences of people dealing with FASD issues in Poland. Since we treated this element of the process as an introductory exercise to open group discussions, we did not assume any levels of acceptance or rejection of individual recommendations. We were more interested in discussing the strengths and weaknesses of each approach and assessing the possibility of introducing similar solutions in Poland.

The responses of Polish specialists clearly indicated that the recommendations of the 4-Digit Code are considered the most useful for assessing growth and dysmorphia. Therefore, the basic problem to be solved in this regard was the selection of appropriate percentile grids for the Polish population. However, for all other elements of the guidelines, the adaptations were more difficult and controversial. First of all, the diagnostic algorithm had to be adapted to the realities of the Polish health service, social assistance, and education. It turned out to be necessary to indicate at least the framework for cooperation between representatives of various professional circles. Moreover, in the area of neurodevelopment, the coordination and integration of psychological, speech therapy, and physiotherapeutic assessment turned out to be problematic due to the lack of a clear distinction between the tasks of each specialist.

We consider the development of the guidelines to be an important step to the elaboration of the Polish system for solving problems related to FASD. However, it is certainly not the end of the tasks we set to ourselves, especially since we are aware of the limitations of our work.

First of all, the guidelines need to be verified in clinical practice. We are aware that it could be challenging to apply the proposed diagnostic procedure in different settings (e.g., hospitals or FASD centers operated by NGOs). The risk that we do acknowledge, is that, unless it is regulated by law, the existing FASD centers will not change the mode of diagnosis to the recommended ones.

A secondary objective of development of the guidelines was to increase the quantity of diagnoses in Poland. An open question is also whether the publication and dissemination of the guidelines (e.g., via trainings addressed not only to specialists in FASD but also to other people working with children, adolescents, and pregnant women) will contribute to an increase in the number of patients referred for FASD diagnosis, which can put the existing system under an unexpected pressure. We also hope that an increased number of the diagnoses made according to the diagnostic guidelines will enable public health estimations essential for planning the healthcare services and political decisions.

Moreover, individual recommendations and/or detailed guidelines contained therein may also require verification. Including the question of the puzzling differences in the most commonly used ICD-10 codes applicable to FASD patients, recommended by specialists in Poland and the American Academy of Pediatrics [62]. The fact that the Polish guidelines lack reference to the *p* codes (certain conditions originating in the perinatal period) is a clear reflection of the fact that, in our country, diagnostics toward FASD is rarely undertaken in newborns. Nevertheless, other differences, e.g., limited use of G (diseases of the nervous system) and R (symptoms, signs, and abnormal clinical and laboratory findings, not elsewhere classified) codes in the Polish system, require further exploration.

The assessment of prenatal alcohol exposure proposed by the Polish team and, in particular the amount of alcohol drunk by a woman during pregnancy, which determines the diagnosis of ND–PAE, should be verified, especially when new research in this field appears. We do not exclude integrating the use of biomarkers to our recommendations; however, there is still too little evidence to ensure their adequate specificity and sensitivity [63,64,65,66]. A controversial aspect of the assessment of PAE concerns the sensitive information about mothers and the possibility of obtaining it from legal, medical, or social services by the diagnostic team. An official inquiry was made to the Personal Data Protection Office (July 2020); however, it was left unanswered to the moment of publication of this article.

There is a dispute about the characteristics of the neuropsychological profile in FASD in the scientific world [67,68]. We are aware that the recommendation regarding neurobehavioral evaluation is predominantly based on the clinical experience of the authors. This recommendation has to be reevaluated as new scientific evidence emerges. Moreover, the recommended test battery should be reassessed with the appearance of new tools available to the Polish population.

We do note that recommended palpebral fissure length charts were not created for the Polish population. We recommend that research aiming at the elaboration of Polish palpebral fissure length charts should be carried out. Similarly, with the availability of new OFC charts or growth charts for European/Polish population, the respective recommendations should be revised.

Notwithstanding the limitations, we believe that Polish guidelines for FASD diagnosis will raise the awareness of the problem among clinicians, teachers, and social workers and will open a new era in this field for the patients’ and their caregivers’ good.

## 5. Conclusions

National and/or regional clinical guidelines for diagnosis of FASD help to standardize diagnosis and to avoid the confusion due to the lack of clear criteria. In order to make recommendations acceptable and useful for national specialists, these have to be elaborated by the interdisciplinary group of professionals representing various groups of stakeholders, taking into account the differences among validated clinical diagnostic systems for FASD in the world, the lack of single biochemical or imaging objective test for FASD, and the specific national healthcare context. Every clinical recommendation requires a periodical evaluation, and an adequate system of their evaluation should be implemented in centers performing the FASD diagnosis.

## Figures and Tables

**Table 1:** A sample question and answer scale.

The Recommendation Is Precise—Clearly Indicates How to Differentiate between Various Sub-Categories of FASD					
	1 I Definitely Do Not Agree	2 I Rather Do Not Agree	3 It Is Hard to Say	4 I Rather Agree	5 I Definitely Agree
IOM guidelines					
Canadian guidelines					
4-Digit Code					
**Substantiation/Comments**					

**Table 2:** Overview of recommended FASD diagnostic criteria.

	FASD		At Risk of FASD—Non-Diagnostic Category
	FAS	ND–PAE	
**Prenatal alcohol exposure**	Yes or unknown	Yes	Yes or, in the presence of 3 sentinel facial features, unknown
**Pre- and/or** **postnatal growth deficits**	Yes	N/A	To be observed
**Sentinel facial features**	- short palpebral fissures and - thin vermilion border of the upper lip and - flat philtrum	N/A	To be observed
**Neurodevelopmental disorders**	- deficits in ≥3 cognitive areas or if neurological symptoms are found in ≥2 cognitive areas and - irregularities in ≥3 areas from the emotional and social sphere, adaptation disorders, or psychopathological symptoms - significant impact of the above-mentioned irregularities in daily life activities and functioning in school, pre-school, or work.		To be observed

## Statement

The full text of the Polish FASD diagnostic guidelines is available at https://www.ciazabezalkoholu.pl/images/Rozpoznawanie_FASD_Pediatria_2020_FASD.pdfy (accessed on 20 May 2021).
